# Incidence of Endodontic Failure Cases in the Department of Conservative Dentistry and Endodontics, DY Patil School of Dentistry, Navi Mumbai

**DOI:** 10.7759/cureus.38841

**Published:** 2023-05-10

**Authors:** Sneha Rao, Vimala Nilker, Manogna Telikapalli, Krupa Gala

**Affiliations:** 1 Conservative Dentistry and Endodontics, D Y Patil (Deemed to be) University School of Dentistry, Navi Mumbai, IND; 2 Public Health, Public Health and Healthcare Quality Professional, New Jersey, USA

**Keywords:** post endodontic restoration, apical periodontitis, peri-apical radiolucency, endodontic failure, root-canal treatment

## Abstract

Introduction: Endodontic and restorative treatment goal is to restore occlusion and normal function of a tooth and provide stability to the dental arch. Root canal bacterial infection and apical periodontitis profoundly impact the management and outcome of endodontic treatments. The crucial goal of nonsurgical root canal therapy (NSRCT) is the mechanical removal of infected tissues and the chemical killing of bacteria. The present study assessed the outcomes and factors associated with the failure of primary endodontic treatment.

Methods: A total of 250 teeth from 219 patients (104 male and 146 female) were examined in the Conservative Dentistry and Endodontics department, who reported symptomatic root canal-treated teeth. Data through clinical examination and radiographic examination was recorded on a proforma designed for the study of each patient regarding endodontic failure.

Results: According to the type of tooth maximum number of teeth that were reported with failure are the molars (67.6%), followed by premolar (14.0%), incisor (12.8%), and lastly, canines (5.6%). Based on the location of affected teeth, the maximum teeth that presented with failed root canal treatment were from mandibular posteriors (51.2%), followed by maxillary posteriors (31.60%), maxillary anterior (13.2%), mandibular anterior (4.0%).

Conclusion: Endodontic failures were mostly found in underfilled root canals and poorly sealed post-endodontic coronal restoration and strong association with peri-apical radiolucency.

## Introduction

The endodontic and restorative treatment goal is to restore occlusion and normal function of a tooth and provide stability to the dental arch. Root canal bacterial infection and apical periodontitis profoundly impact the management and outcome of endodontic treatments. The link between bacterial infection of the root canal system and apical periodontitis has had a tremendous impact on endodontic clinical strategies directed toward its management [1].

The crucial goal of nonsurgical root canal therapy (NSRCT) is the mechanical removal of infected tissues and the chemical killing of bacteria [1]. Obturation creates a dense three-dimensional fill of canal space and accessory canals, preventing residual bacteria growth or intrusion of new bacteria [1]. A dense fill of this nature terminated at the appropriate working length has been shown clinically to produce superior results. Obturation fulfilling these criteria helps push the successful initial healing of NSRCT to 76%-86% [2,3].

There are two categories mentioned in literature trying to quote the outcomes of endodontic therapy: survival and success. Endodontic success is defined as an absence of clinical symptoms and a reduction in pre-existing lesion size. Survival or functional retention is known as tooth retention with asymptomatic function. Here an asymptomatic endodontically treated tooth may be associated with a radiolucency yet deemed successful [4]. Strindberg believed endodontic post-treatment sequelae require an asymptomatic patient with no periapical radiolucency; otherwise, cases are deemed failures [5]. 

An endodontically treated tooth should be clinically and radiographically evaluated for success. Patients should be scheduled for follow-ups to ascertain that the treatment is successful and that the tooth is functional. A myriad of factors has been implicated in the failure of endodontic treatment. The usual factors which can be attributed to endodontic failure are the persistence of bacteria, inadequate filling of the canal, overextensions of root-filling materials, improper coronal seals, untreated canals, iatrogenic procedural errors, and complications of instrumentation. Despite these complexities, root canal therapy is extremely cost-effective and has allowed the retention of teeth.

Our observational study aimed to analyze factors and outcomes associated with the failure of primary endodontic treatment, reporting in the Conservative Dentistry and Endodontics department. Also, to study the factor most and least associated with failed primary root canal treatment.

## Materials and methods

The present study was an observational cross-sectional in-vivo study done in the Conservative Dentistry and Endodontics department. Over twelve months, 219 patients from October 2018 to September 2019 reported to the Conservative and Endodontics department with primary endodontic failures. The sample size was calculated based on the 95 percent power where the number of individuals with root canal therapy in the particular population was determined and taken prevalence from a previous report by a study [2]. The Institutional Ethical Committee approval was taken, and informed consent from patients was obtained with ethical number FRC/2018/Cons/06. After taking demographic information like age, gender, occupation, and medical history, a thorough clinical evaluation of teeth and adjoining structures was made.

Patients having permanent dentition with good oral hygiene were included in the study. The study did not include patients with cyst enucleation, vertical root fracture, apicoectomy, non-restorable teeth, and third molars. Two periapical radiovisiographs were taken, with straight angulation and Clark's rule. Two evaluators systematically examined all the radiographs. The analysis's high-quality radiographs allowed for the evaluation of each tooth's apex, apical region, and level of root filling. Every radiograph was methodically inspected and the evaluation was done.

**Figure 1:**
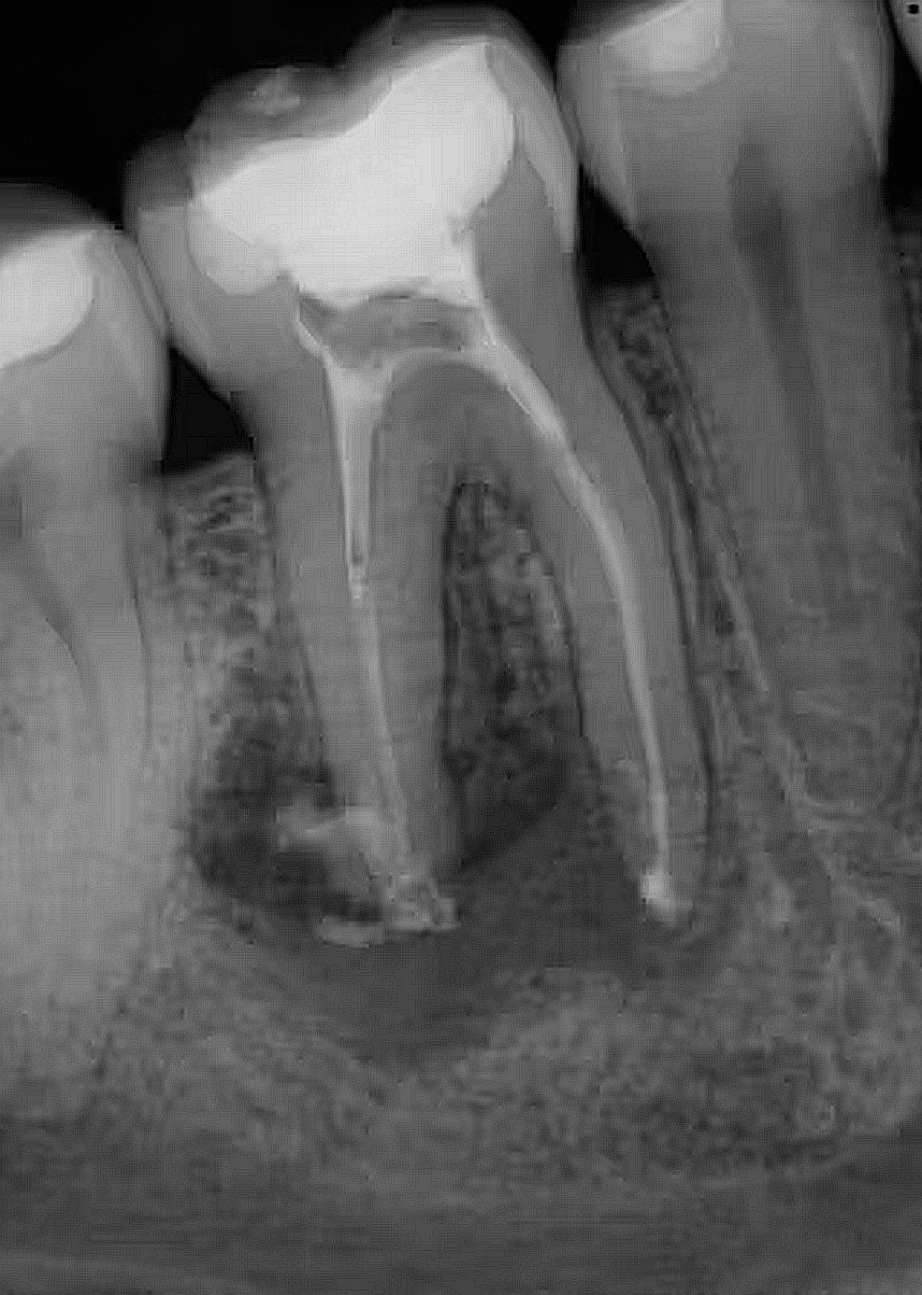
Failed endodontic therapy

Data for endodontic treatment failure was entered onto a proforma created for the study. Age and sex, tooth type, the number and location of teeth with apical lesions, the technical quality of root canal fillings (the length of the filling from the radiographic apex), presentations during clinical examinations, associated periapical radiolucency, and procedure errors were all noted on a structured form for each subject (Figure 1).

Evaluation standards were as follows: 1) Radiopaque substance in a tooth's pulp chamber or root canal. Technically, root canal fillings can be classified as acceptable (filling 0-2 mm short of the radiographic apex) or inadequate (filling >2 mm short, protruding past the radiographic apex, or limited to the pulp chamber). 2) The apical portion of the periodontal ligament should not be wider than the lateral periodontal ligament space to maintain a healthy periodontal ligament, or it may be radiolucent at the root apex if it is wider than the lateral periodontal ligament. 3) Whether post and core are present or not. 4) Procedure Errors (Instrument separation, ledges, or perforation). 5) Obturation quality (Is the filling homogeneous, or are there any voids?).

The Chi-square correlation test was applied using Excel and R-programming software. The Statistical Package for Social Sciences (SPSS) for Windows 26.0 was used to enter and analyze the data. The 95 percent confidence intervals were used, and a p-value of 0.05 was considered statistically significant.

## Results

According to the type of tooth maximum number of teeth that were reported with failure were molars (67.6%), followed by premolar (14.0%), incisors (12.8%), and lastly, canines (5.6%). According to affected teeth location, the maximum number of teeth that presented with failed root canal treatment were from mandibular posteriors (51.2%), followed by maxillary posteriors (31.60%), maxillary anterior (13.2%), mandibular anterior (4.0%). Maximum failure cases presented after 1-3 years (30.4%) of primary root canal treatment were due to pulpal disease inflicted by dental caries (88.0%). Most teeth presented without full coverage restoration (75.2%), but its relation to periapical health was not statistically significant (Table 1, Figure 1).

**Table 1:** Presentations during clinical examinations

Presentations during clinical examinations	Frequency (N)	Percentage (%)
Pain	25	10.0%
Pain + Pain on percussion	28	11.2%
Pain + Sinus tract	1	0.4%
Pain on percussion	165	66.0%
Pain on percussion + Pain on percussion with swelling	1	0.4%
Pain on percussion + Sinus tract	3	1.2%
Pain on percussion with swelling	21	8.4%
Pain on percussion with swelling and sinusitis	5	2.0%
Sinus tract	1	0.4%

**Figure 2:**
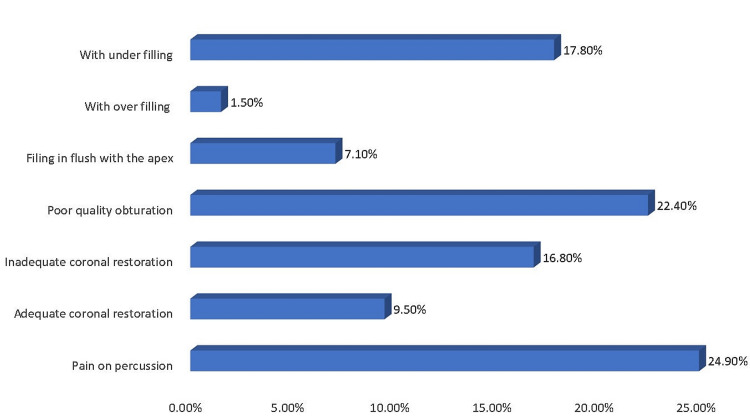
Associated periapical radiolucency

Out of 250 teeth, maximum teeth presented with inadequate coronal restoration with respect to the quality of seal and margins (overhangs or deficient) when evaluated radiographically (79.6%) as well as through clinical assessment (58.4%). Maximum failure cases presented with filling 3mm or shorter of the radiographic apex (47.70%). The presence of voids, space between canal wall and obturating material, absence of homogenous density, and inadequate lateral condensation were significantly associated with failed root canal-treated teeth in our study (p=0.001). Inadequate canal preparation blocked the apical end of the canal, and ledges were significantly associated with failed root canal-treated teeth in our study (P=0.001). Out of 250 teeth, periradicular radiolucency was noted in maximum teeth (62.8%) (Figure 2).

## Discussion

In the study, the mean age was 36.29±12.28 years, ranging from 13 to 88 years. 41.6% of males and 58.4% of females were in the study group. Thus, females presented with maximum failures than their male counterparts. Ng et al., in their systemic review, showed reduced tooth survival after root canal treatment for male patients compared to female patients [6]. In this study, all the 250 teeth examined exhibited one or more clinical signs and symptoms, such as spontaneous pain or pain on percussion, sinus tract opening, or swelling. Patients with signs of endodontic failures but asymptomatic were excluded from the study. Out of 250 teeth, a higher percentage of failure was noted in molars (67.6%), followed by premolars (14.0%), incisors (12.8%), and the least in canine (5.6%). The study exhibited multi-rooted teeth with a maximum percentage (74.4%) of failure than those with single roots (25.6%). In the Noboru Imura et al. study, multirooted teeth exhibited a reduced success percentage than premolars and anterior teeth [7].

The Chi-square correlation test revealed obturation defects such as voids, space between canal wall and obturating material, absence of homogenous density, and inadequate lateral condensation was significantly maximum in molars (P<0.05). Procedural defects such as inadequate canal preparation and ledges are significantly maximum in molars. (P<0.05). Akbar et al. also noted maximum failure in molars compared to other teeth, especially the mandibular molars (41%) [8]. It was noted that the maximum failure reported to the location of the affected teeth was in mandibular posteriors (51.2%), followed by (31.6%) in maxillary posteriors, (13.2%) maxillary anterior, and (4.0%) in mandibular anterior teeth. It was noted that the canines showed the lowest percentage of failure compared to the other type of teeth, and failure was recorded more in the mandibular than in the maxillary arch.

However, Ng et al. noted that teeth excluding molars had a greater survival rate after endodontic therapy [6]. Vire et al. concluded that the ratio of failure of mandibular teeth to maxillary teeth was 7:3 [9]. In Olcay et al.'s study, the maxillary teeth (52.7%) showed a higher percentage than mandibular teeth in a sample size of 1000 endodontically failed patients. (47.3%) [10]. Of the 250 teeth, 220 teeth were treated endodontically due to disease of the pulp, the primary reason being dental caries (88%), followed by trauma (7.2%), other non-carious lesions of the dentition (2.8%) and intentional root canal therapy was done for about 2% of the cases. Our study findings are similar to Ng et al.'s since intentional root canal therapy presented with minimum failure. In contrast, the probability of the spread of infection does increase in other conditions [6]. According to Quality guidelines for endodontic treatment, the peri-apical lesion should be assessed for at least 4 years until it has resolved. If it persists after 4 years of root canal treatment, it is found to be associated with post-treatment disease and is considered an unfavorable outcome [11].

Since our study consisted only of symptomatic cases, patients presented with teeth as early as 6 months post initial root canal treatment up to 5 years after treatment. Maximum (30.4%) teeth presented with failure after 1-3 years of completion of treatment. Salehrabi et al. found that treatment relapse mainly occurred within 3 years after the completion of primary root canal treatment [12]. It was noted that the most prevalent sign of failure presented was pain on percussion (66.0% ), followed by (11.2%) of teeth with spontaneous pain along with pain on percussion, (10.0%) of teeth with spontaneous pain, (8.4%) of teeth with pain on percussion and swelling, (2.0%) teeth with pain on percussion along with swelling and sinus opening, (1.2%) teeth with pain on percussion along with sinus tract, pain on percussion with swelling and sinus tract being present in the same frequency of single case (0.4%). Lin et al., in their histologic study, found an association between stainable bacteria inside the canal with swelling and pain or a draining sinus tract [13].

In the survey study, 78.8% of failed teeth had post-endodontic restoration, followed by 21.2% of teeth that did not have post-endodontic coronal restoration. Imura et al. identified the absence of restoration; as one of the predictors of endodontic outcome. He inferred that the absence of permanent restoration positively correlated with endodontic failure [7]. During clinical examination, maximum failure was noted in teeth with inadequate coronal restoration (58.4%), followed by teeth with adequate coronal restoration (41.6%). Also, during the radiographic assessment of failure cases, maximum teeth (79.6%) had inadequate coronal restoration. Tsesis et al. found that poor coronal restoration adversely affects periapical lesion long-term prognosis [14]. Ricucci et al., in their study in 2011, found that coronal restoration quality does not affect treatment outcomes. He also found that excess root canal-filling material reduced success [15].

It was noted that a maximum number of teeth with failed root canal treatment were obturated with root filling ending >3 mm from the radiographic apex (47.7 %), followed by teeth obturated with root filling ending </=3 mm from the radiographic apex (30.2%), teeth with obturations in flush with radiographic apex (19.1%), 3% teeth obturated with over-filling. Iqbal et al. found that underfilled canals (33.3%) were most responsible for endodontic failures [16]. Ng et al., in their systemic review, found a higher survival probability for teeth with flush root fillings than teeth with short root fillings [17]. Segura-Egea et al., in a study, concluded that 73.9% of teeth with an inadequate adaptation of filling were associated with apical periodontitis [18]. In our study, an inadequate adaptation of the filling and lateral condensation was the most common factor associated with failure to the quality of obturation. A higher percentage of cases presented with periapical radiolucency (62.8%), followed by teeth without periapical radiolucency (37.2%). The main reason for apical periodontitis persistence after endodontic treatment is bacteria penetrating the root canal due to coronal leakage. Tsesis et al., in a study, stated that poor root canal filling (61%) and poor restoration (48%) adversely affected periapical status dynamics [14]. Our study noted a significant finding since maximum failed root canal-treated teeth were reported with no full coverage restoration. Salehrabi et al. study revealed that 85% of teeth did not have full crown coverage. The number of failures leading to teeth extraction with no crown was 4.8-fold higher in anterior teeth, 5.8-fold higher in premolars, and 6.2-fold higher in molars [12]. The maximum percentage of failure was in roots with multiple canals (76.0%), followed by roots with single canals (24.4%). In the Indian population using computed tomography, Prakash et al. concluded that three canals were present in 84.48% of mandibular first molars, with 13.52% having four canals [19]. We aimed to analyze the level of motivation when more than one failure was noted in the dentition. However, interestingly, the maximum number of patients were willing to retreatment (96.4%) [20].

The limitation of the study was that the patients were in a limited area, and regarding the department collected and the sample size was small.

## Conclusions

Maximum primary root canal treatment failure was noted in molars and least in canine, in the mandibular arch than the maxillary arch, and in teeth without full-coverage crown. Failures were primarily recorded 1-3 years after primary endodontic treatment completion. Quality of obturation is a prognostic factor determining endodontic treatment outcome. Narrow canals were the least common factor associated with failed root canals. Inadequate canal preparation in blocked canals due to ledges is most commonly seen in failed endodontically treated teeth. Within the limitations of our study, endodontic treatment failures mostly occurred in underfilled root canals and poorly sealed post-endodontic coronal restoration, along with association with peri-apical radiolucency.
